# Conformational plasticity underlies membrane fusion induced by an HIV sequence juxtaposed to the lipid envelope

**DOI:** 10.1038/s41598-020-80156-w

**Published:** 2021-01-14

**Authors:** Igor de la Arada, Johana Torralba, Igor Tascón, Adai Colom, Iban Ubarretxena-Belandia, José L. R. Arrondo, Beatriz Apellániz, José L. Nieva

**Affiliations:** 1 Instituto Biofisika (CSIC-UPV/EHU), University of the Basque Country (UPV/EHU), PO Box 644, 48080 Bilbao, Spain; 2 Department of Biochemistry and Molecular Biology, University of the Basque Country (UPV/EHU), PO Box 644, 48080 Bilbao, Spain; 3 Ikerbasque, Basque Foundation for Science, 48013 Bilbao, Spain; 4 Department of Physiology, Faculty of Pharmacy, University of the Basque Country (UPV/EHU), Paseo de la Universidad, 7, 01006 Vitoria-Gasteiz, Spain

**Keywords:** Membrane structure and assembly, Membrane biophysics

## Abstract

Envelope glycoproteins from genetically-divergent virus families comprise fusion peptides (FPs) that have been posited to insert and perturb the membranes of target cells upon activation of the virus-cell fusion reaction. Conserved sequences rich in aromatic residues juxtaposed to the external leaflet of the virion-wrapping membranes are also frequently found in viral fusion glycoproteins. These membrane-proximal external regions (MPERs) have been implicated in the promotion of the viral membrane restructuring event required for fusion to proceed, hence, proposed to comprise supplementary FPs. However, it remains unknown whether the structure–function relationships governing canonical FPs also operate in the mirroring MPER sequences. Here, we combine infrared spectroscopy-based approaches with cryo-electron microscopy to analyze the alternating conformations adopted, and perturbations generated in membranes by CpreTM, a peptide derived from the MPER of the HIV-1 Env glycoprotein. Altogether, our structural and morphological data support a cholesterol-dependent conformational plasticity for this HIV-1 sequence, which could assist cell-virus fusion by destabilizing the viral membrane at the initial stages of the process.

## Introduction

**Figure 1:**
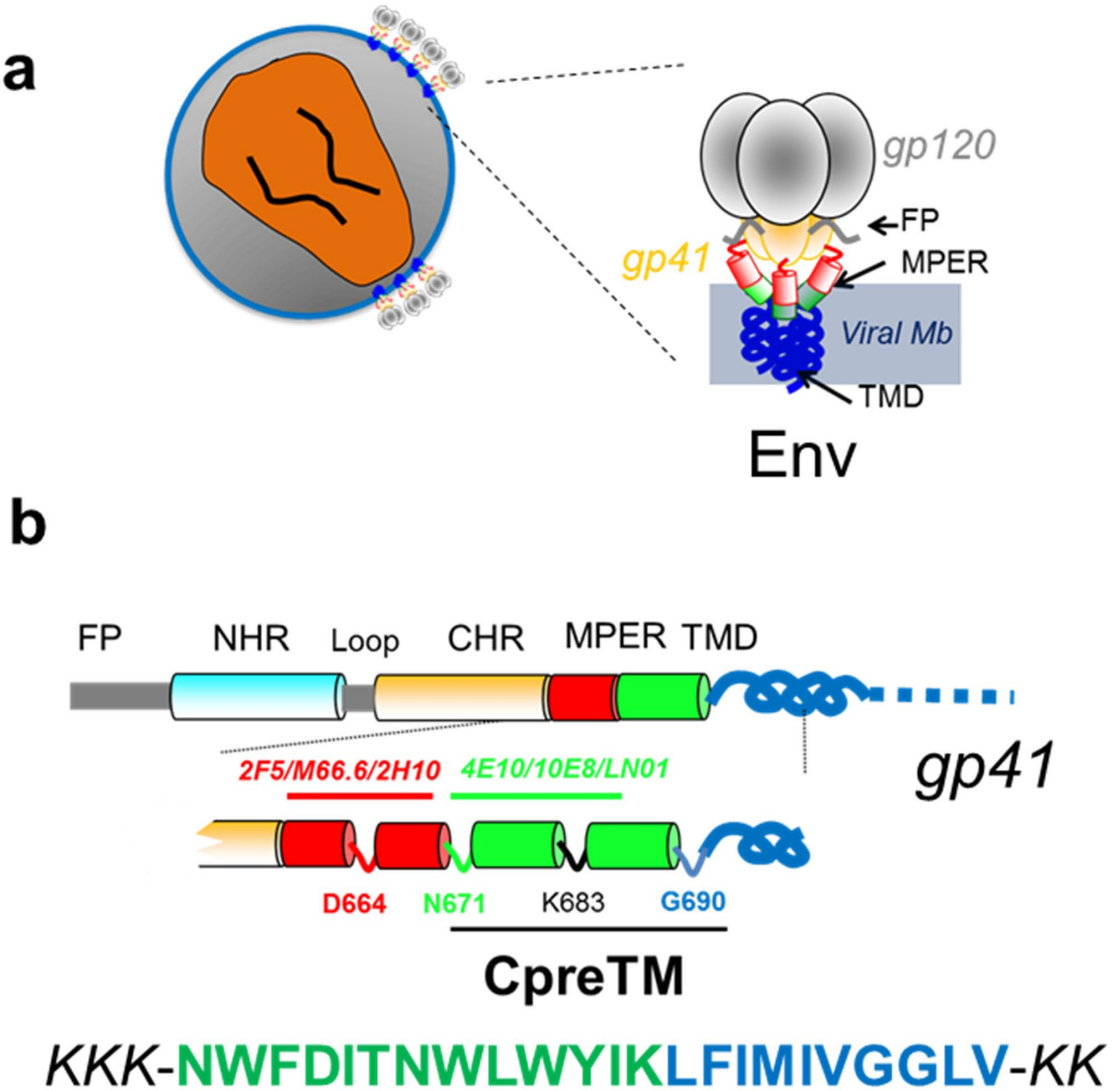
Designation of the HIV-1 CpreTM sequence used in this study. (**a**) Schematic displaying the general organization of the HIV-1 Env glycoprotein in virions (pre-fusion state). (**b**) Diagram showing the constituents of the gp41 subunit ectodomain and transmembrane anchor. Functional domains designated within its sequence include FP, fusion peptide; NHR and CHR, amino- and carboxy-terminal helical regions, respectively; MPER, membrane-proximal external region; TMD, transmembrane domain (see also Supplementary Fig. S1). The MPER-TMD region contains epitopes for the recognition of several broadly neutralizing HIV antibodies as indicated. The CpreTM sequence that derives from this region is shown below.

During the early phase of the replication cycle, the Human Immunodeficiency Virus type-1 (HIV-1) particle fuses its lipid envelope with the plasma membrane of the CD4^+^ target cell^1,2^. The reaction is triggered after engagement of the envelope glycoprotein (Env) with the cell receptor (CD4)/co-receptor (CXCR4 or CCR5), a specific recognition process that activates further refolding of the metastable native Env. Fusion activity of Env depends on the presence of the fusion peptide (FP), a conserved sequence located at the N-terminus of the transmembrane subunit gp41 (reviewed in Ref.^3,4^). Following fusion triggering, the FP is propelled towards the target cell membrane and embeds therein due to its hydrophobic character^1,2^. Subsequently, helical regions within gp41 ectodomains refold into an energetically stable, trimeric 6-helix bundle (6-HB), whose formation brings together the merging membranes: the plasma membrane of the target cell, and the lipid envelope of the virus (Fig. 1, see also Supplementary Fig. 1)^5–7^.

In analogy to FPs, insertion into the viral membrane of a C-terminal gp41 sequence known as the Membrane-Proximal External Region (MPER), is postulated to further contribute to the overall process of membrane merger (Fig. 1, see also Supplementary Fig. 1)^8–13^. This sequence, sitting at the bottom of the Env complex is exceptionally enriched in aromatic residues that promote interactions with the membrane interface (reviewed in Ref.^14–17^). MPER insertion may be disruptive for the viral membrane, since peptides and constructs derived from its sequence have been shown to exert virucidal effects^18–21^. The discovery that a number of antibodies targeting MPER can block membrane activity and infection, further underlines the importance of this region for the fusogenic function of the Env glycoprotein (reviewed in Ref.^16,22^).

There is structural evidence to support that the carboxy-terminal MPER sequence can combine with transmembrane domain (TMD) residues of gp41 to form a continuous helix, at least in one of the conformational states that are accessible to the pre-fusion Env complex^23–30^. A peptide that spans this helix (CpreTM, Fig. 1b), has been shown to induce lipid bilayer restructuring upon partitioning into cholesterol (Chol)-enriched virus-like membranes^21,31–33^. However, it remains to be established whether structure–function relationships displayed by the standard N-terminal FP of gp41, also apply to this MPER-derived C-terminal sequence.

Cumulative experimental work using synthetic surrogates and model membranes has delineated physiologically relevant aspects of the FP function (reviewed in Ref.^4,34,35^). High-resolution NMR studies reveal the adoption of continuous α-helical structures in membrane mimics^36,37^, whereas the combination of Infrared (IR) and Solid-State Nuclear Magnetic Resonance (SS-NMR) spectroscopy demonstrates that the α-helical conformation can convert into oligomeric β strand structures, a transition promoted by peptide density and the increase of the Chol concentration in the membrane^38–40^. Attenuated Total Reflectance (ATR)-IR studies further indicate that monomeric FP α-helices penetrate into lipid bilayers in an oblique angle^41,42^, while its oligomeric β-strand counterparts appear to associate with the main axis forming a 90º angle with respect to the membrane normal^43^.

Thus, regarding its conformational behavior, a hallmark of membrane-bound HIV FP appears to be its plasticity, which enables the transition from inserted α-helices, tilted relative to the bilayer normal, into extended β-strands, lying almost parallel to the membrane plane^3,4,39–41,43–47^. Studies in model systems suggest that these alternating conformations of the FP sequence can disrupt the lipid bilayer, breaching its permeability barrier and/or inducing aggregation and inter-bilayer mixing of lipids^40,44,47–50^. Moreover, the membrane-inserted FP appears to modulate the elastic properties of the bilayer and facilitate the formation of the non-bilayer lipid intermediates required for fusion^51–54^. In this context, the HR1 and HR2 helical domains within the ectodomain of gp41 are conceived as a mechanical device that brings the host-cell plasma membrane, primed for merger by the inserted FP, into contact with the viral membrane (Supporting Fig. S1).

In this work, we combine conventional IR spectroscopy, two-dimensional correlation IR spectroscopy (2D-COS-IR), and ATR-IR, to analyze the conformation and orientation adopted by the CpreTM peptide upon reconstitution into lipid bilayers. In line with the notion that the sequences flanking the TMD anchors of fusion glycoproteins are endowed with a degree of conformational plasticity^55–57^, our data reveal that the CpreTM helix can adopt membrane-inserted α-helical structures that convert primarily into an extended β-strand conformation in Chol-rich membranes. Occurrence of the extended conformation lying parallel to the membrane plane correlates with the induction of vesicle fusion as visualized by cryo-electron microscopy (cryo-EM) of vitrified specimens. Thus, we conclude that CpreTM bound to membranes displays structural features of canonical FPs, and propose a structure-based mechanistic model that couples CpreTM helix unfolding to membrane merger during the HIV-1 fusion cascade.

## Results

### CpreTM conformation in a low-polarity medium

**Figure 2:**
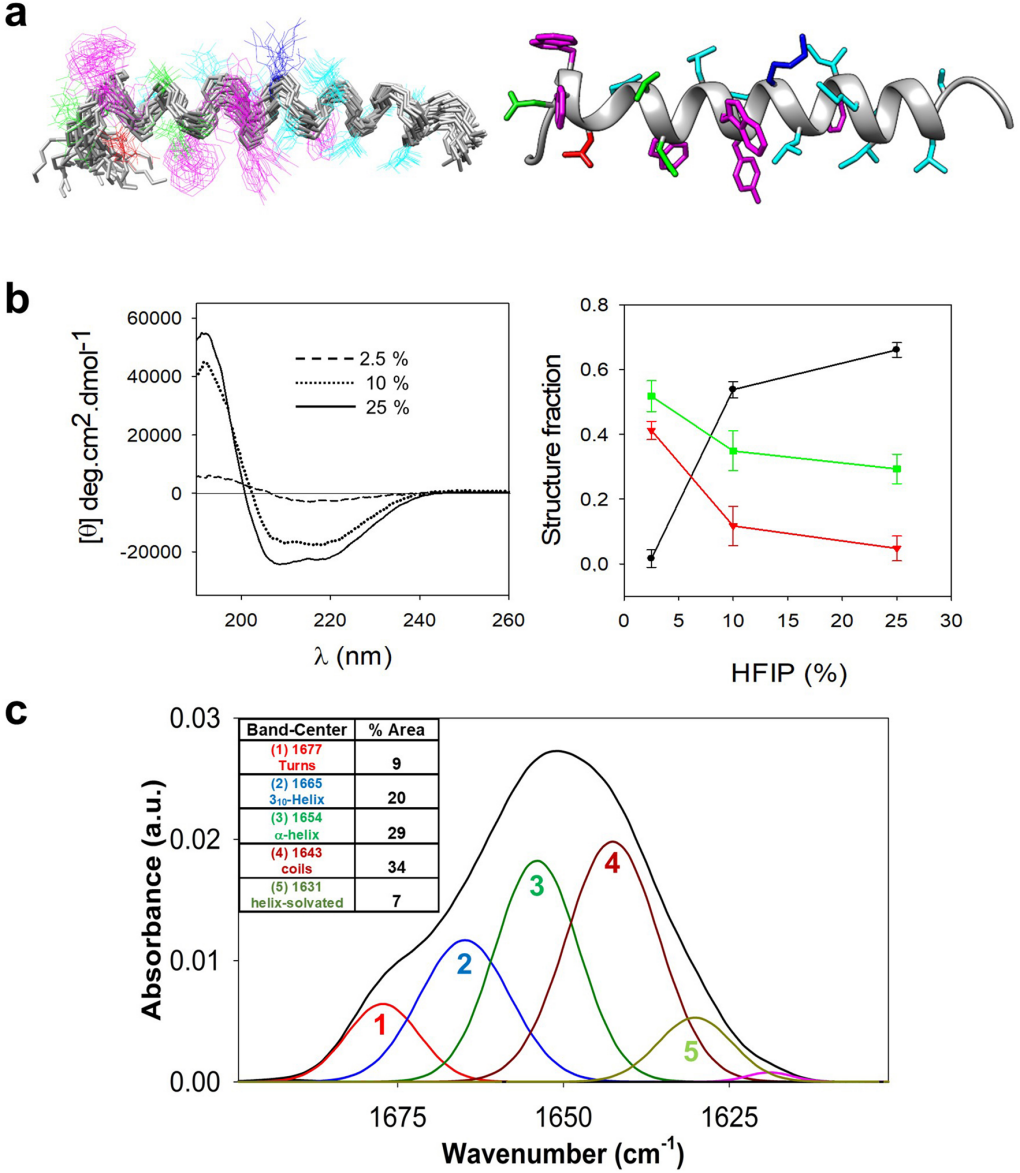
Structural analysis in a low-polarity medium. (**a**) NMR structure of the CpreTM peptide solved in 25% 1,1,1,3,3,3-hexafluoro-2-propanol (HFIP) (v/v) (PDB ID: 2MG2). (**b**) Left: CD spectra obtained at 25 °C in media containing increasing amounts of HFIP as indicated in the panel; Right: secondary structure fractions estimated from CDPro^83^. Means ± the standard deviation for the fraction values estimated with CONTIN-LL, CDSSTR, and SELCON3 are plotted. Black dots, red triangles and green squares depict the fractions of helix, strand and the sum of turns + unordered conformations, respectively. (**c**) IR spectrum in the amide I region obtained in buffer containing 25% HFIP (v/v). The absorption band was decomposed into different components. The original spectrum and the sum of the band components are superimposed and indistinguishable. The inset displays the secondary structure assignation for the main components (bands labeled with numbers 1 to 5) and the area percentages (rounded off to the nearest integer).

Before establishing the membrane-bound conformations of the CpreTM peptide, we analyzed the secondary structure adopted in a medium that mimics the low-polarity of lipid bilayers. As a reference we employed the published NMR structure of monomeric CpreTM in buffer containing 25% (v/v) 1,1,1,3,3,3-hexafluoro-2-propanol (HFIP)^24^. Figure 2a displays the superposition of the calculated models for the CpreTM structure in HFIP, and a representative single model (left and right panels, respectively). All calculated models were consistent with a predominantly α-helical geometry, with evidence for disordered regions limited to the COOH and NH_2_ extremities. Panel b displays the circular dichroism (CD) spectra obtained for CpreTM in buffers with increasing HFIP content, and the quantitative analysis of the secondary structure composition^58^ (left and right panels, respectively). Consistent with peptide aggregation in solution, β-strands and turn/coil structures dominated the spectra at the lowest HFIP concentration (2.5% v/v), and diminished upon decreasing polarity. At the highest HFIP concentration where monomers are expected to be favored (25% v/v), the α-helix contribution was predominant (ca. 65%), whereas only a residual signal from peptide aggregation remained. In these samples, components attributable to the disordered conformations and turns amounted to ca. 30%.

Matching those findings, band decomposition of IR spectra obtained in 25% HFIP identified a majority of amide-I vibrational modes arising from helical conformers, with bands centered at 1665 cm^−1^ (3_10_-helix), 1655 cm^−1^ (α-helix) and 1630 cm^−1^ (α-helix solvated)^59–61^ amounting to ca. 56% of the total band area (Fig. 2c). Besides, a band centered at 1642 cm^−1^ (ca. 34%) was ascribed to disordered coil structures, whereas that at 1677 cm^−1^ was attributed to turns (ca. 9%). Again, consistent with the monomeric state of the peptide (Panel a), the contribution of extended-aggregated conformations was negligible in these samples (< 1%).

### CpreTM conformation in lipid bilayers

**Figure 3:**
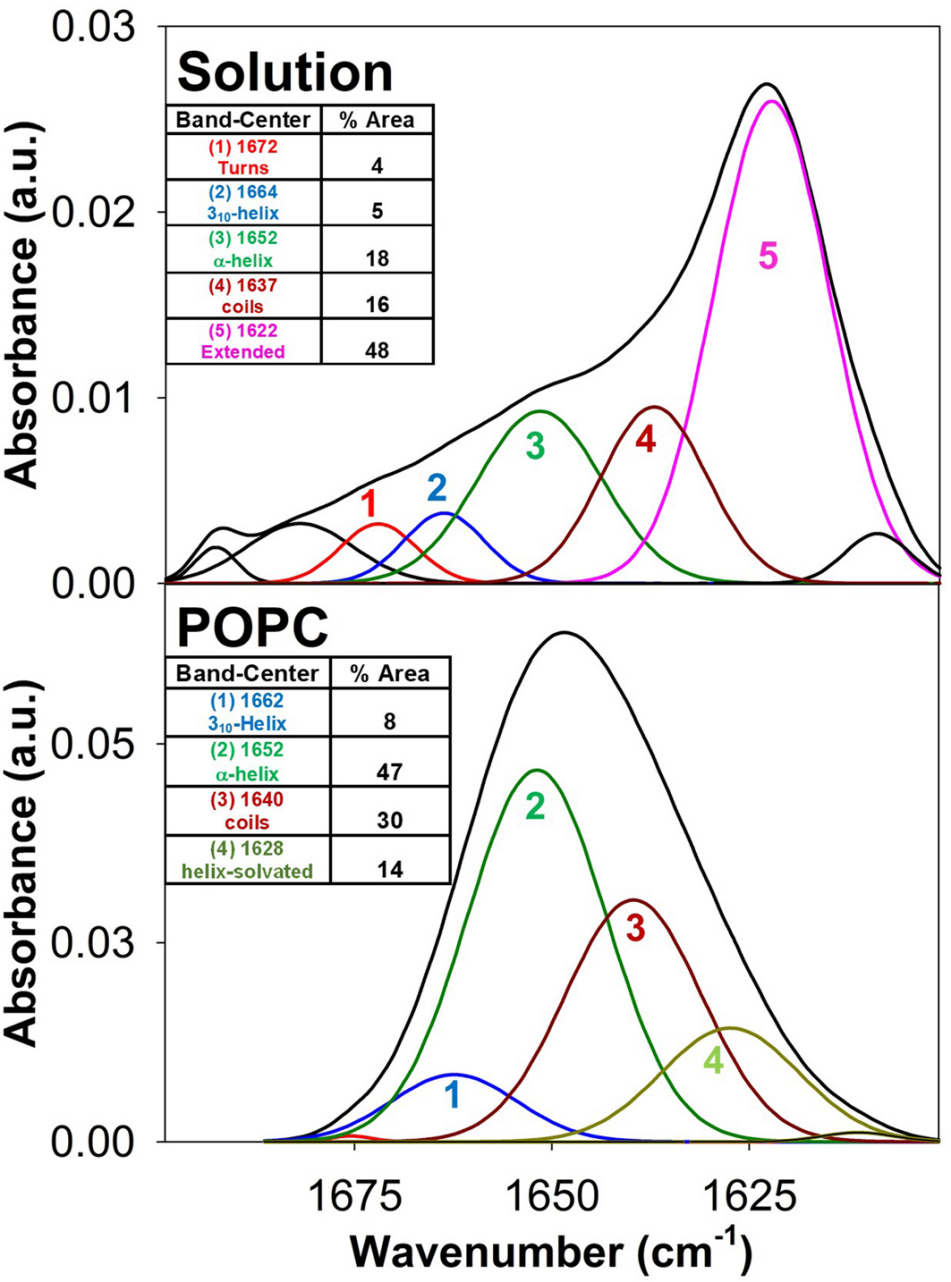
Reconstitution of CpreTM in lipid bilayers. Top and bottom IR spectra display respectively the amide I components measured in buffer or after reconstitution of the peptide in POPC lipid bilayers (Peptide-to-lipid ratio, 1:50). Insets in both panels display the secondary structure assignation for the main band components and their area percentages.

We next reconstituted CpreTM in membranes by co-mixing it with lipids in organic solvent, followed by gentle evaporation and hydration (see Materials and Methods). Figure 3 compares the IR spectrum of CpreTM in solution with that obtained after reconstitution in lipid bilayers made of 1-palmitoyl-2-oleoyl-*sn*-glycero-3-phophocholine (POPC) (top and bottom panels, respectively). The amide-I region of the IR spectrum in solution displayed a prominent band centered at 1622 cm^−1^, which together with high-frequency absorption in the 1680–1690 cm^−1^ region, denoted that a majority of peptide chains were unfolded/aggregated. In contrast, upon reconstitution in POPC bilayers, the maximum shifted to 1654 cm^−1^, whereas the contribution of the 1620 cm^−1^ band was irrelevant. In these samples predominant helical conformers amounted to ca. 70%. Besides, in comparison with the absorption band components measured in HFIP (Fig. 2c), the contribution of turns and disordered chains decreased, whereas the amide-I band became overall narrower. These spectral variations reflect a reduction in the conformational space accessible to the CpreTM chain upon reconstitution in lipid bilayers, consistent with the majority of the membrane-associated peptide adopting a canonical α-helical conformation.

### Conformational changes in cholesterol-containing membranes

**Figure 4:**
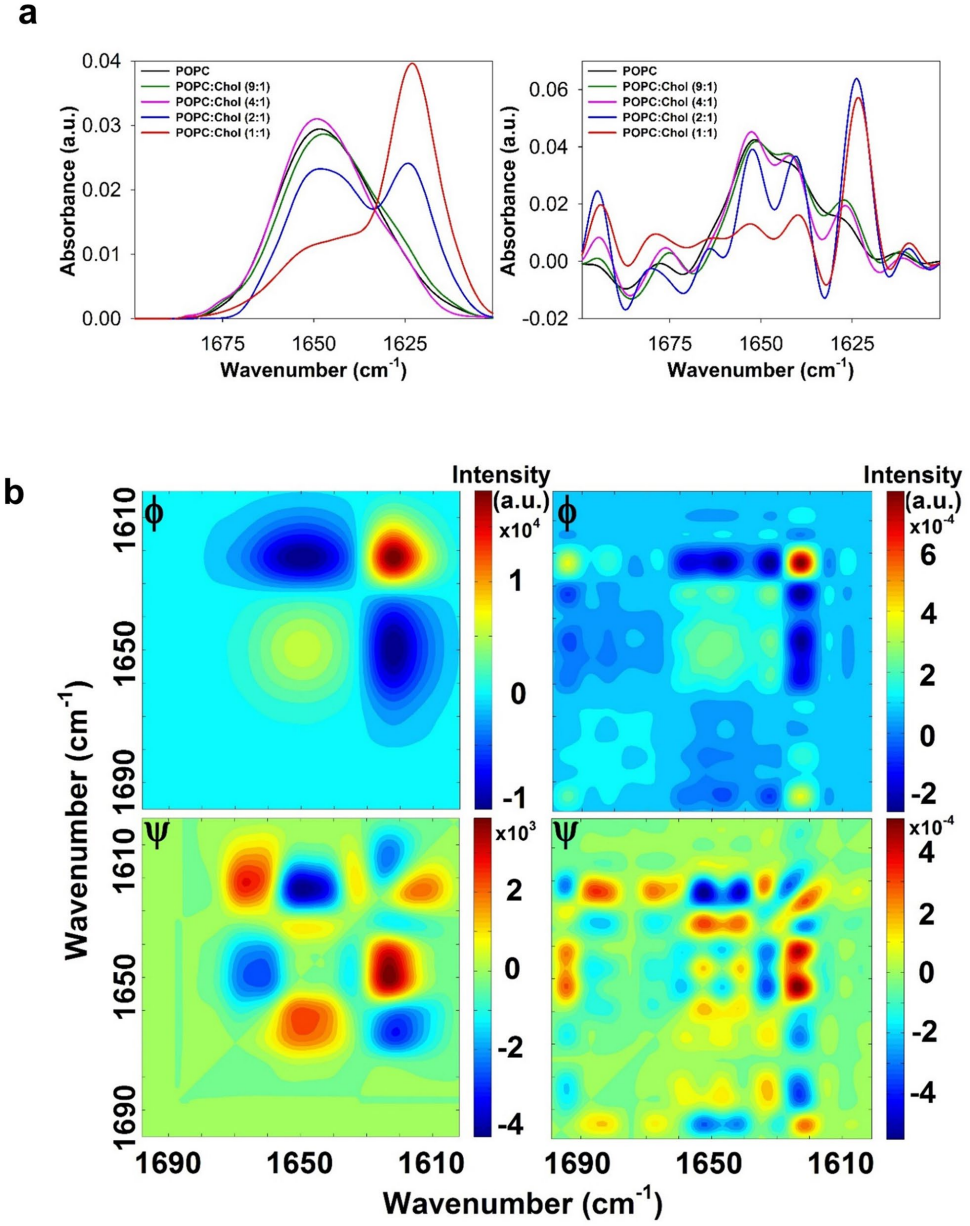
Conformations adopted by CpreTM as a function of the Chol content in membranes. (**a**) Amide I region IR spectra of CpreTM reconstituted in membranes (peptide-to-lipid ratio, 1:50) that contained increasing Chol concentrations as indicated in the panels. Raw and deconvolved spectra are shown (left and right panels, respectively). (**b**) 2D-COS IR analysis. Synchronous (top) and asynchronous (bottom) correlation map contours of the raw (left) and deconvolved (right) IR spectra obtained with increasing Chol concentrations are shown. Red peaks correspond to positive correlations and blue peaks to negative ones.

Cholesterol (Chol) is a major lipid of the HIV membrane required for virion infectivity^62–66^. Therefore, we analyzed the conformation adopted by CpreTM reconstituted in membranes containing increasing Chol concentrations. Figure 4a displays the series of raw and deconvolved IR spectra as a function of Chol content in membranes (left and right panels, respectively). A shoulder centered at ca. 1620 cm^−1^ could be already discerned in the samples that contained low Chol, which evidenced an initial accumulation of extended chains. Samples containing the highest Chol concentrations displayed a more conspicuous band centered at 1622 cm^−1^, consistent with a β-strand-like conformation dominating the secondary structure of the membrane-bound peptide under these conditions.

To get more insight into the CpreTM conformational changes induced by the membrane Chol concentration, we next performed the 2D-correlation analysis of the IR spectra in the corresponding amide I band region^67–69^ (Fig. 4b). We note that relevant effects detected on the 2D maps often reflect subtle changes in the relative contents of the amide I band components. Therefore, in addition to the β-strand band that dominates in samples containing high concentrations of Chol (centered at ca. 1620 cm^−1^), the analysis also revealed the evolution of the rest of the spectral components i.e., bands centered at ca. 1675, 1660, 1650, 1642 and 1635 cm^−1^.

In the synchronous (Φ) 2D maps of CpreTM (Fig. 4b, top panels), autopeaks indicated simultaneous changes in the bands composing the amide-I spectrum. In the 2D maps of the raw spectra (left), autopeaks were found centered at 1650 and 1620 cm^−1^, whereas the single cross-relation negative peak 1620/1650 cm^−1^ reflected that both vibrations were affected in-phase by Chol, the first component augmenting in intensity, the second diminishing. Higher resolution was attained using the Φ map based on the deconvolved spectra (right). Particularly, all helical components were evidenced as autopeaks centered at ca. 1635 cm^−1^, 1655 cm^−1^ and 1665 cm^−1^, which could be observed together with cross-relation negative peaks 1620/1635 cm^−1^, 1620/1655 cm^−1^ and 1620/1665 cm^−1^.

The corresponding asynchronous (Ψ) maps reflected the sequential order of events induced by the increase of Chol (Fig. 4b, bottom panels)^67–69^. The asynchronous peaks were positive (red contours) if the change in the first frequency occurred accelerated with respect to that in the second one, and negative (blue contours) if delayed. The positive correlation peak 1655/1665 cm^−1^ detected in the raw-spectra maps, suggests the formation of less stable short regions deviating from canonical α-helicity and adopting 3_10_-helical geometries, whereas the negative one at 1620/1665 cm^−1^ supports the conversion of the 3_10_-helix intermediates into extended strands.

This pathway was also apparent in the Ψ map based on deconvolved spectra. In this case, an additional positive correlation peak was found for the pair 1635/1665 cm^−1^. It is known that partial solvation of α-helical structures can give rise to low-frequency bands centered at ca. 1635–1630 cm^−1^ because of the cross-hydrogen bonds that can be formed with water^60,61^ (see also Fig. 2c). Thus, we attribute the CpreTM absorption mode at 1635 cm^−1^ to a fraction of the helical structure not buried in the membrane, i.e., exposed to solvent and/or in contact with interfacial polar moieties. The positive correlation found at 1635/1665 cm^−1^ suggests that these solvated helices also unfold adopting 3_10_-helical geometries, whereas the negative one at 1635/1655 cm^−1^ would be consistent with the buried helical fraction unfolding more readily than the solvent-exposed one upon increasing the Chol concentration.

Additional positive peaks were observed at 1655/1675 cm^−1^ and 1635/1675 cm^−1^, and a negative peak found at 1620/1675 cm^−1^. This indicates that β-turns can also act as intermediates of the helix-to-strand unfolding process. In conclusion, upon increasing the Chol content in the membrane, β-turns/3_10_-helical regions seem to be produced at the expense of the canonical α-helical conformations, and these intermediates appear to convert into extended strands.

To establish whether the C-terminal region accounted for the tendency of MPER to adopt extended conformations in membranes, we also analyzed the effect of Chol on the conformation adopted by NpreTM, a peptide overlapping with the aromatic-rich N-terminal stretch of CpreTM, but lacking its TMD residues (Supplementary Fig. S2a). Following CpreTM’s trend, NpreTM reconstituted in POPC membranes adopted a main helical conformation (Supplementary Fig. S2b). However, when reconstituted in POPC:Chol (1:1) membranes, β-strands did not dominate the overall conformation of the NpreTM peptide, supporting the implication of the membrane-buried CpreTM TMD residues in the conformational conversion promoted by Chol.

### Membrane insertion angle of alternating conformations

**Figure 5:**
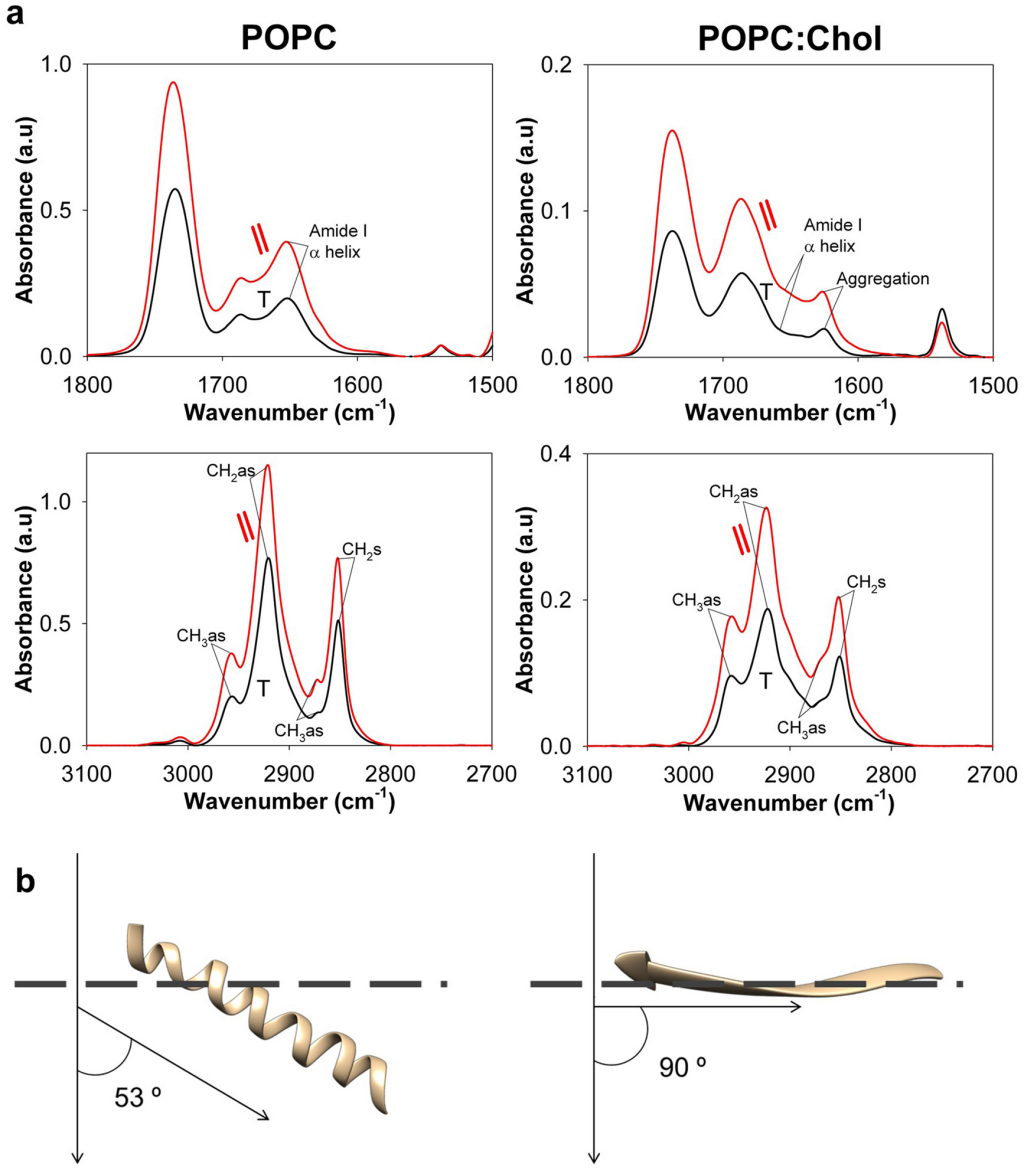
Angle of insertion of CpreTM main conformations as determined by ATR-IR. (**a)** Top: Comparison of ATR-IR spectra in the amide I region of CpreTM reconstituted in POPC or POPC:Chol (1:1) membranes (left and right panels, respectively) (peptide-to-lipid mole ratio, 1:50). Bottom: a similar comparison was made in the CH_2_/CH_3_ stretching region of the spectra. The main orientations of peptide and acyl chains are inferred from the ratio of peak areas recorded with incident light polarized parallel (||) and perpendicular (┬) to the membrane normal (calculated values for the order parameters and tilt angles are displayed in Table 1). (**b**) Models for the membrane-associated structures and orientations adopted by CpreTM in POPC and POPC:Chol (1:1) membranes (left and right panels, respectively).

The previous results support the efficient lipid bilayer reconstitution of the CpreTM peptide as an α-helix, and the possibility of its transitioning to extended structures in Chol-enriched membranes. Using ATR-IR spectroscopy, we next determined the tilt of these alternating CpreTM conformations relative to the membrane normal. ATR-IR absorbance spectra were measured using perpendicular and parallel polarized light (Fig. 5a). From these spectra, the experimental average dichroic ratios were determined, and order parameters *S* and tilt angles calculated (Table 1)^70–72^.

**Table 1:** ATR-IR data of the CpreTM peptide.

Wavenumber (cm^−1^)	Vibration^a^	θ (º)^b^	Avg dichroic ratio ± SD	Avg *S* ± SD^c^	Avg γ ⊥± SD^d^	Avg ^L^ ± SD^e^
Lipid alone (POPC)						
2920	*as* CH_2_ stretching	90	1.33 ± 0.00	0.48 ± 0.00	35.97 ± 0.15	
2850	*s* CH_2_ stretching	90	1.19 ± 0.00	0.62 ± 0.00	32.28 ± 0.20	
2870	*s* CH_3_ stretching	0	9.57 ± 0.30	0.69 ± 0.01	27.05 ± 0.39	
CpreTM + POPC						
2920	*as* CH_2_ stretching	90	1.50 ± 0.01	0.34 ± 0.01	41.60 ± 0.15	
2850	*s* CH_2_ stretching	90	1.47 ± 0.01	0.36 ± 0.01	40.68 ± 0.38	
2870	*s* CH_3_ stretching	0	5.88 ± 0.33	0.53 ± 0.02	34.07 ± 0.86	
1656	Amide I–helix	30	2.03 ± 0.03	0.02 ± 0.01	54.13 ± 0.55	53.27 ± 1.32
Lipid alone (POPC:Chol)						
2920	*as* CH_2_ stretching	90	1.62 ± 0.02	0.24 ± 0.01	45.21 ± 0.44	
2850	*s* CH_2_ stretching	90	1.50 ± 0.00	0.34 ± 0.00	41.53 ± 0.04	
2870	*s* CH_3_ stretching	0	1.49 ± 0.04	-0.17 ± 0.02	61.95 ± 0.75	
CpreTM + POPC:Chol						
2920	*as* CH_2_ stretching	90	1.69 ± 0.01	0.20 ± 0.01	47.06 ± 0.20	
2850	*s* CH_2_ stretching	90	1.62 ± 0.01	0.25 ± 0.01	44.99 ± 0.38	
2870	*s* CH_3_ stretching	0	3.18 ± 0.72	0.24 ± 0.12	45.50 ± 4.55	
1623	Amide I–sheet	70	2.42 ± 0.15	-0.33 ± 0.11	71.55 ± 6.82	90.00 ± 0.00

^a^Vibrations are presented as symmetric (*s*) or asymmetric (*as*).

^b^, θ direction of the dipole moment associated with the vibration with respect to the direction of the main molecular axis (aliphatic chain or peptide-secondary structure).

^c^*S*, form factor.

^d^γ⊥, angle between the direction of the molecular axis and the perpendicular to the crystal plane (similar to the membrane plane).

^e^γ^L^, angle between the direction of the peptide-secondary structure axis and the calculated aliphatic chain axis.

According to the tilt angle inferred from the dichroic ratios, the longitudinal axis of the CpreTM helix formed an angle of 53º with the POPC lipid bilayer normal (Fig. 5b). Angles of a comparable magnitude (ca. 50º) have been reported in the literature for the HIV-1 and SIV FPs inserted into lipid bilayers^42,46^. Thus, our ATR-IR data support that, similarly to the N-terminal FP, the C-terminal Env sequence covered by CpreTM could insert in a tilted orientation into the membrane at some stage during the fusion process. Conversely, the CpreTM β-strands oriented almost parallel to the membrane plane in the POPC:Chol (1:1) sample (angle of ca. 90º with respect to the normal) (Fig. 5b), also in accordance with data reported in the literature for the N-terminal FP under fusogenic conditions^39,43,44^.

### Changes in vesicle morphology induced by CpreTM reconstituted in membranes

**Figure 6:**
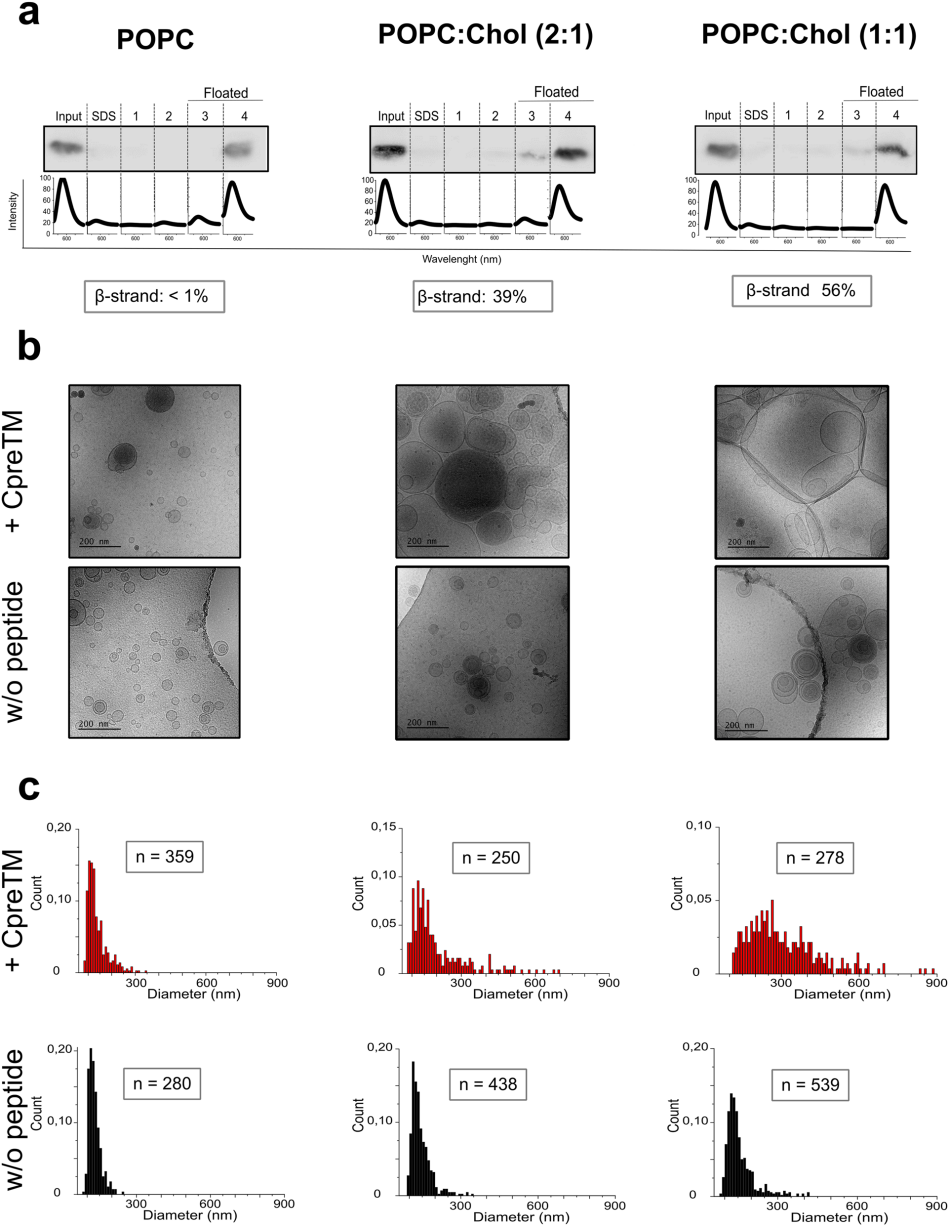
Vesicle morphology changes induced by the different conformations adopted by CpreTM in membranes. (**a**) Vesicle flotation analysis. Incorporation to vesicles of CpreTM was verified after ultracentrifugation in a sucrose gradient (peptide-to-lipid mole ratio, 1:50). The presence of the peptide in the floated and non-floated fractions of the gradient and in the original sample (input) was revealed by Western Blot analysis after Tris-Tricine SDS-PAGE separation. Fluorescence emission spectra below reveal the presence of Rhodamine-labeled vesicles in the collected fractions. The amount of extended β-strand adopted by the peptide in the samples is indicated below the panels (values determined in the spectra of Fig. 5a after band decomposition). (**b**) Vesicle morphology by cryo-EM. Images obtained for vesicles containing reconstituted peptide are shown in the top-panels. Bottom panels display images of vesicles devoid of peptide. (**c**) Vesicle size distribution as determined from the diameters measured in cryo-EM images. The sizes of oval-shaped vesicles were determined by measuring their major axes. Conditions as those in the previous panel.

Despite the differences in the attained conformation, the reconstituted CpreTM peptide incorporated to the same extent and quantitatively into vesicles containing different concentrations of Chol (Fig. 6a). In contrast, Cryo-EM images revealed different morphologies for the peptide-containing vesicles, suggesting that the adopted conformations induced distinct patterns of membrane destabilization (Fig. 6b,c and Supplementary Fig. S3). Untreated control samples displayed spherical vesicles with heterogeneous sizes ranging in mean diameter from ca. 100 to 200 nm (Fig. 6b,c, bottom panels). The α-helical peptide did not alter the overall morphology or size of POPC vesicles when incorporated at a 1:50 peptide-to-lipid dose (Fig. 6b,c, top left panels). In contrast, an increase of extended conformations in the peptide-treated samples (Chol-containing membranes) correlated with a significant increase of the mean vesicle size (Fig. 6b,c, top center and right panels). Particularly, in peptide-containing POPC:Chol (1:1) samples, massive aggregation and vesicle sizes in the range of 500 nm could be observed.

The Supplementary Figure S3 displays more detailed views of the effects exerted by CpreTM on vesicle morphology. The peptide reconstituted in POPC membranes did not affect the stability of the vesicle samples, whereas its inclusion into POPC:Chol membranes induced tight vesicle-vesicle contacts (lipid bilayer aggregation) and increased the mean diameter of the vesicles (membrane fusion). Notably, POPC:Chol (1:1) vesicles containing the reconstituted NpreTM control peptide displayed a pattern of tight bilayer aggregation, which did not result in an increase of the mean vesicle size (Supplementary Fig. S2c). Thus, it appears that completion of the fusion process required the presence of the CpreTM TMD residues.

Lipid aggregates with spongy morphology also accumulated sporadically in certain areas of CpreTM-containing POPC:Chol vesicles (Supplementary Fig. S3, bottom panels). The occurrence of lipid aggregates reminiscent of non-lamellar arrangements suggested that, following an FP-like fashion^4,51–54^, the CpreTM peptide could also facilitate the formation of highly curved lipid structures involved in membrane merger^35^. However, control experiments using ^31^P-NMR failed to detect evidence for the promotion of this type of non-lamellar arrangements in peptide-containing POPC:Chol (1:1) membranes (Supplementary Fig. S4). Moreover, an inspection by Atomic Force Microscopy (AFM) of supported lipid bilayers containing reconstituted peptide revealed that CpreTM disrupted the lipid continuity of the solvent-accessible membrane monolayer, and increased the amount of force required to break the bilayer (Supplementary Fig. S5). Thus, it appears that inclusion of CpreTM at doses leading to vesicle fusion did not facilitate membrane deformation by increasing curvature or reducing bilayer stiffness^35^.

## Discussion

**Figure 7:**
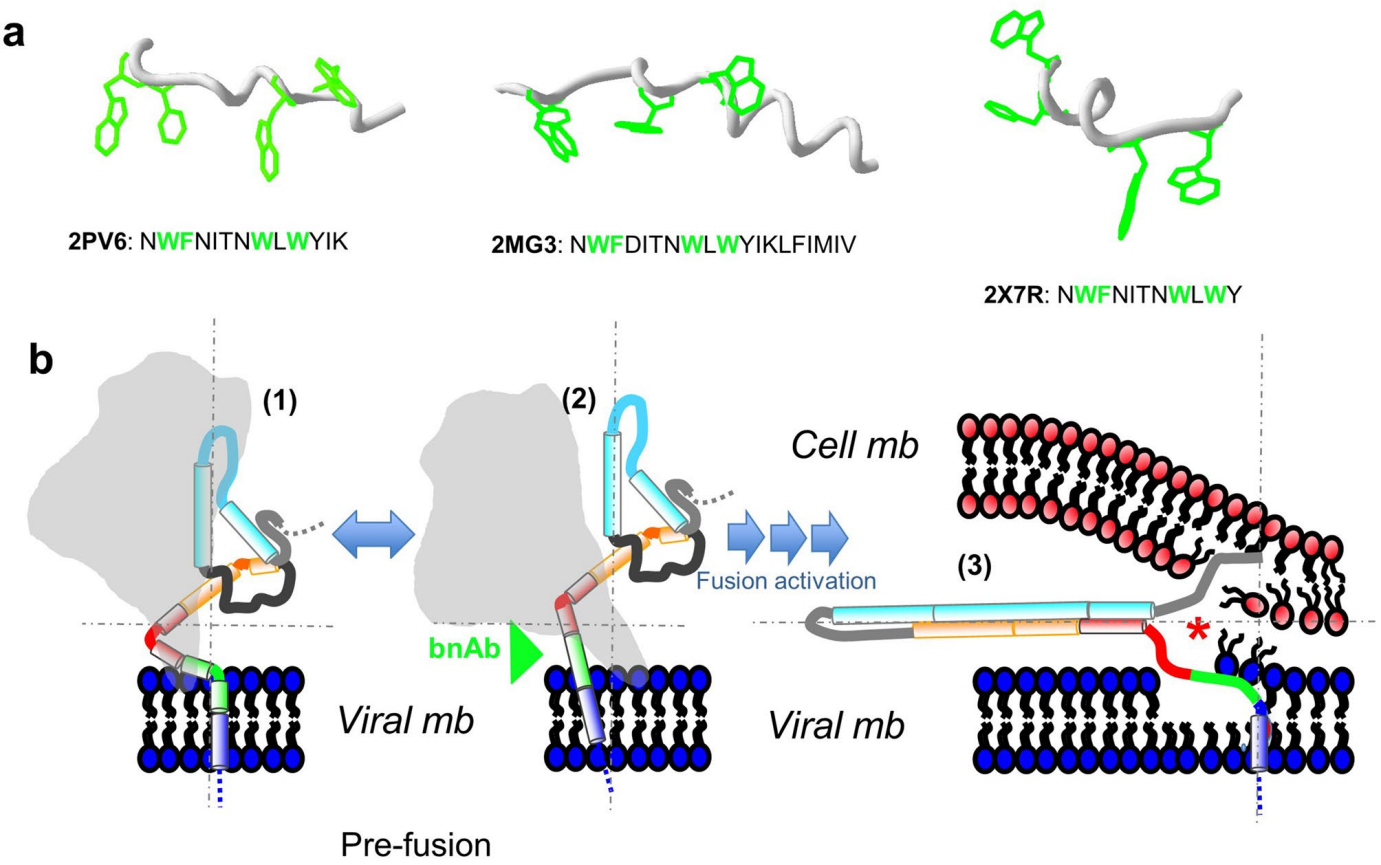
Proposed role for CpreTM sequence in an extended conformation during the process of Env-mediated HIV fusion. (**a**) High-resolution structures of MPER sequences displaying sections of extended chain conformation. PDB accession numbers used to render the panel together with the sequence ranges covered are displayed below. (**b**) Mechanistic model: Compact (1) and open (2) conformations of the Env glycoprotein are postulated to interchange spontaneously before engagement with receptor/co-receptor (one Env monomer is depicted for simplicity). The cartoon representing the gp41 subunit is based on the pre-fusion X-ray structure (PDB accession code: 4TVP) and illustrates the relative positions of the most important constituents in the ectodomain (same color code as in previous Fig. 1). Upon activation of the fusion cascade, helical regions reposition to interact with each other initiating the assembly of the 6-HB, whereas the main axis of the complex align with the membrane plane (3). This process is proposed to be facilitated by the extension of the MPER chain connecting the enlarging 6-HB to the TMD. Insertion of the MPER connection into the viral membrane in an extended conformation may prime it for fusion (*), creating poorly solvated spots to facilitate initial interbilayer contacts and/or generating a lipid bridge between the merging membranes.

Studies in model systems support a predominant α-helical conformation for monomeric forms of the membrane-bound HIV FP, which appear to insert tilted relative to the membrane normal^42–44,48,73^. In addition, the membrane-inserted FP helix can undergo conformational changes leading to the formation of extended β-strands, which have been associated with the perturbation of the lipid bilayer architecture and the promotion of lipid mixing during membrane fusion^40,43,44,48–50,74,75^. Such conformational plasticity would be at odds with the stagnant α-helical conformation generally assumed for the MPER-TMD sequences of gp41 in the context of the Env glycoprotein^24,27–29,76–78^. However, challenging the existence of a single MPER conformation after biogenesis, several studies support that a substantial portion of the membrane-embedded MPER can exist in an extended conformation during the gp41 refolding process that accompanies fusion activation (Fig. 7a)^7,24,79^. Epitope peptides resolved in complex with antibody fragments also suggest that partly extended MPER chains can be targeted by certain antibodies^80,81^. In a more general sense, it has been argued that sequences flanking viral glycoprotein TMD helices might unfold adopting β-strand conformations, and contribute to the promotion of the fusion process by imparting negative curvature to the bilayer^55^.

Here, we employed IR spectroscopy approaches to analyze the conformations accessible to the membrane-proximal CpreTM sequence reconstituted in membranes. Together, the spectroscopy results confirm that the reconstitution process results in a membrane-inserted CpreTM α-helix, which is partially exposed to solvent and orients in an oblique angle with respect to the membrane plane. Chol appears to induce conformational changes leading to the formation of β-strands that lie mainly parallel to the membrane plane. The occurrence of CpreTM extended chains is associated with the destabilization of the lipid bilayers, as suggested by cryo-EM imaging and AFM characterization.

The model displayed in Fig. 7b integrates these findings in a general model of HIV-1 gp41-induced membrane fusion. The prefusion Env complex may alternate compact (1) with more open conformations (2), and it is likely that in these structures the helices spanning the MPER-TMD sequence could kink at different positions. Recent structural studies support that, at least in one of those conformational states, a straight and continuous CpreTM helix inserted in a subtle angle would be the target to antibodies exerting broad and potent neutralization^29,30^.

Subsequent activation of the fusion process involves the refolding of the gp41 helical domains HR1 and HR2 (depicted in cyan and yellow colors, respectively) to initiate the formation of a compact 6-HB. Establishment of the extensive helix-helix hydrophobic interactions between HR1 and HR2 implies the relocation of the helical sections and the reorientation of the complex main axis with respect to the membrane (3). We infer that the initial formation of the 6-HB likely requires extension of the Cα chain at sections joining the emerging complex to the membrane-inserted sequences. These extended hydrophobic chains, most prominently at FP and MPER areas, could associate with membrane surfaces helping to overcome repulsive hydration and electrostatic forces, as the cell and viral membranes approach pulled by the growing 6-HB hairpin. Furthermore, our data suggest that the CpreTM sequence could also break lipid continuity of the viral membrane external monolayer (Supplementary Fig. S5), generating poorly solvated hydrophobic spots where the initial contacts could be established between the approaching bilayers.

Overall, the experimental data presented in this study support the notion that a similar conformational plasticity underpins the membrane activity of the FP and CpreTM region during the initial (and transient) stages of HIV-1 fusion, but caution that effects of these sequences on the elastic properties of membranes involved in the process might be different. In this regard, we note that the present work provides no hints as to how the membrane-inserted structures of the FP or MPER evolve at later stages of the fusion process. It has been argued that the FP could first assemble as β-sheets on membrane surfaces, but later convert into α-helices to complete fusion^38^. Thus, at least theoretically, it is possible that at later stages of the fusion process the CpreTM sequence also attains secondary structures and membrane topologies that differ from those described in this work. Such alternative conformations might allow completion of the 6-HB structure and/or modulate the elastic properties of the membrane to facilitate fusion^54^.

## Materials and methods

### Reagents

The peptide sequence derived from the gp41 MPER-TMD region, *KKK*-NWFDITNWLWYIKLFIMIVGGLV-*KK* (CpreTM) (Fig. 1) was produced by solid-phase synthesis using Fmoc chemistry as C-terminal carboxamides and purified by HPLC (estimated purity 97%). 1-palmitoyl-2-oleoyl-*sn*-glycero-3-phophocholine (POPC) and cholesterol (Chol) were purchased from Avanti Polar Lipids (Birmingham, AL, USA). N-(lissamine Rhodamine B sulfonyl) phosphatidylethanolamine (N-Rh-PE) was from Thermo Fisher Scientific (Waltham, Massachusetts, USA). 1,1,1,3,3,3-hexafluoro-2-propanol (HFIP) was obtained from Sigma-Aldrich (St. Louis, Missouri, USA). Monoclonal antibody 4E10 (MAb4E10), kindly donated by D. Katinger (Polynum Inc., Vienna, Austria), and rabbit anti-human-IgG-HRP (Santa Cruz Biotechnologies) were used to reveal the membrane-bound peptide.

### CpreTM reconstitution in membranes

To prepare CpreTM-containing vesicles, adequate amounts of lipids and peptide were mixed in organic solvent prior to the production of the liposomes as described^82^. Briefly, phospholipid and cholesterol were dissolved in chloroform:methanol 1:2 (vol:vol) and mixed with CpreTM (dissolved in 100% ethanol) at a peptide-to-lipid molar ratio of 1:50. The mixture was dried under a N_2_ stream followed by 2 h vacuum pumping to remove traces of organic solvents. Subsequently, the dried lipid films were subjected to 2 h of gentle hydration with H_2_O using a N_2_ gas bubbler to facilitate dispersion of the dried lipid-peptide film in PBS buffer. Next, the multilamellar vesicles were bath sonicated (1 h, 55 °C) and subjected to 15 freeze and thaw cycles to obtain unilamellar vesicles. Finally, effective incorporation of the peptide to the vesicles was ensured by peptide flotation coupled to that of lipid vesicles after ultracentrifugation of the samples in a sucrose gradient as described^32^.

### Circular dichroism

Circular dichroism (CD) measurements were carried out on a thermally-controlled Jasco J-810 circular dichroism spectropolarimeter calibrated routinely with (1S)-( +)-10-camphorsulfonic acid, ammonium salt. CpreTM stock samples dissolved in DMSO, were lyophilized and subsequently dissolved in an aqueous buffer (2 mM Hepes, pH, 7.4) at 0.03 mM concentration with 2.5%, 10% or 25% (v:v) 1,1,1,3,3,3-hexafluoro-2-propanol (HFIP). Spectra were measured in a 1 mm path-length quartz cell equilibrated at 25 °C. Data were taken with a 1 nm band-width, 100 nm/min speed, and the results of 20 scans per sample were averaged. Quantitative analysis of the spectra was carried out using the CDPro software^83^, as previously described^58^.

### Transmission infrared spectroscopy

Infrared spectra were recorded in a Thermo Nicolet Nexus 5700 (Thermo Fisher Scientific; Waltham, MA) spectrometer equipped with a mercury-cadmium-telluride detector using a Peltier based temperature controller (TempCon, BioTools Inc., Wauconda, IL) with calcium fluoride cells (BioCell, BioTools Inc., Wauconda, IL). CpreTM-containing samples were lyophilized and subsequently prepared at 3 mg (peptide)/mL in D_2_O buffer (PBS). A 25 μl sample aliquot was deposited on a cell that was sealed with a second cell. Reference windows without peptide were prepared similarly. Typically 370 scans were collected for each background and sample, and the spectra were obtained with a nominal resolution of 2 cm^−1^. In the HFIP samples solvent contribution was subtracted from the original spectra before the data analysis to allow a reliable comparison between spectra.

Data treatment and band decomposition of the original amide I have been described elsewhere^59^. In brief, the number and position of bands were obtained from the deconvolved (bandwidth = 18 and k = 2) and the Fourier derivative (power = 3 and breakpoint = 0.3) spectra. The baseline was removed before starting the fitting procedure and initial heights set at 90% of those in the original spectrum for the bands in the wings and for the most intense component, and at 70% of the original intensity for the rest of bands. An iterative process followed, in two stages. (i) The band position of the component bands was fixed, allowing widths and heights to approach final values; (ii) band positions were left to change. For band shape a combination of Gaussian and Lorentzian functions was used. The restrictions in the iterative procedure were needed because initial width and height parameters can be far away from the final result due to the overlapping of bands, so that spurious results can be produced. In this way, information from band position, percentage of amide I band area and bandwidth were obtained for every component. Using this procedure the result was repetitive. Mathematical accuracy was assured by constructing an artificial curve with the parameters obtained and subjecting it to the same procedure again. The number of bands was fixed on the basis of the narrowing procedures. The molar absorption coefficient for the different bands was assumed to be similar and within a + / − 3% error.

To obtain the 2D-COS-IR maps, the Chol content was used to induce spectral fluctuations and to detect dynamic spectral variation in the secondary structure of CpreTM. Rendering of the two-dimensional synchronous and asynchronous spectra has been described previously^69^.

### Attenuated total reflection IR spectroscopy (ATR-IR)

ATR-IR spectra were measured in a Bruker Tensor 27 spectrometer equipped with a mercury-cadmium-telluride detector using a BioATRCell II micro-ATR unit. 20 μl of the lipid mixtures containing peptide were dried on the surface of the ATR Ge crystal by flowing dried air into the infrared spectrometer chamber during 5 h. For spectra acquisition, the polarized mirror (Pike Technologies) was adjusted to 0° and 90°, to generate incident light oriented parallel and perpendicular to the lipid normal, respectively. 150 IR spectra at 2 cm^−1^ resolution were collected under each condition and averaged. The dichroic ratio of the amide I bond absorption was computed for parallel (0°) versus perpendicular (90°) polarized incident light relative to the membrane normal and was employed to calculate the peptide orientation as discussed previously^71,84,85^.

### Electron microscopy

As initial screen to determine the optimal concentration, samples were first imaged by negative stain electron microscopy. 8 μL aliquots were adsorbed onto glow-discharged carbon coated copper grids, and negatively stained with 1% uranyl formate. Specimens were imaged with a JEM-1230 transmission electron microscope (JEOL Ltd. Tokyo) using an Orius SC1000 (4008 × 2672 pixels) cooled slow-scan CCD camera (Gatan Inc.) at the equivalent nominal magnification of 20000x, and defocus values between -2 and -5 µm. Selected samples were then vitrified and imaged using a JEM-2200FSC transmission electron microscope (JEOL Ltd. Tokyo) equipped with a field emission gun (FEG) operated at 200 kV and an in-column omega energy that helped us to record images with improved signal-to-noise ratio (SNR) by zero-loss filtering, using an energy selecting slit width of 30 eV centered at the zero-loss peak of the energy spectra. Digital images were recorded under low dose conditions using a 4 K x 4 K UltraScan 4000™ charge-coupled device (CCD) camara (Gatan Inc.) at the equivalent nominal magnification of 50000x, and defocus values between -1.5 and -4 µm. 4 μL aliquots were applied onto Quantifoil R 2/2 on 300 mesh cooper grids and C-flat R 1.2/1.3 on 400 mesh cooper grids plasma cleaned with air for 5 s using a PDC-002-CE plasma cleaner (Harrick Plasma). Grids were blotted and plunge frozen in liquid ethane with an automated Leica EM GP2 automatic plunge freezer (Leica Microsystems GmbH, Wetzlar).

## Supplementary Information

Supplementary Information.
