# Supplementary material for: Conformational plasticity underlies membrane fusion induced by an HIV sequence juxtaposed to the lipid envelope
Source: Sci Rep. 2021 Jan 14;11:1278. doi: 10.1038/s41598-020-80156-w (PMC7809034; doi:10.1038/s41598-020-80156-w)
Supplement: Supplementary file 1 — Supplementary Information. [file 41598_2020_80156_MOESM1_ESM.pdf]

Supplementary Information

Conformational plasticity underlies membrane fusion induced by an HIV sequence juxtaposed to the lipid envelope

Igor de la Arada[1&], Johana Torralba[1,2&] , Igor Tascón[1,3], Adai Colom[1,2,3], Iban Ubarretxena-Belandia[1,3], José L. R. Arrondo[1,2], Beatriz Apellániz[4] and José L. Nieva[1,2*]

[1]Instituto Biofisika (CSIC-UPV/EHU), University of the Basque Country (UPV/EHU), PO Box 644, 48080 Bilbao, Spain.

[2]Department of Biochemistry and Molecular Biology, University of the Basque Country (UPV/EHU), PO Box 644, 48080 Bilbao, Spain.

[3]Ikerbasque, Basque Foundation for Science, 48013, Bilbao, Spain

[4]Department of Physiology, Faculty of Pharmacy, University of the Basque Country (UPV/EHU), Paseo de la Universidad, 7, 01006 Vitoria-Gasteiz, Spain.

Supplementary Figures

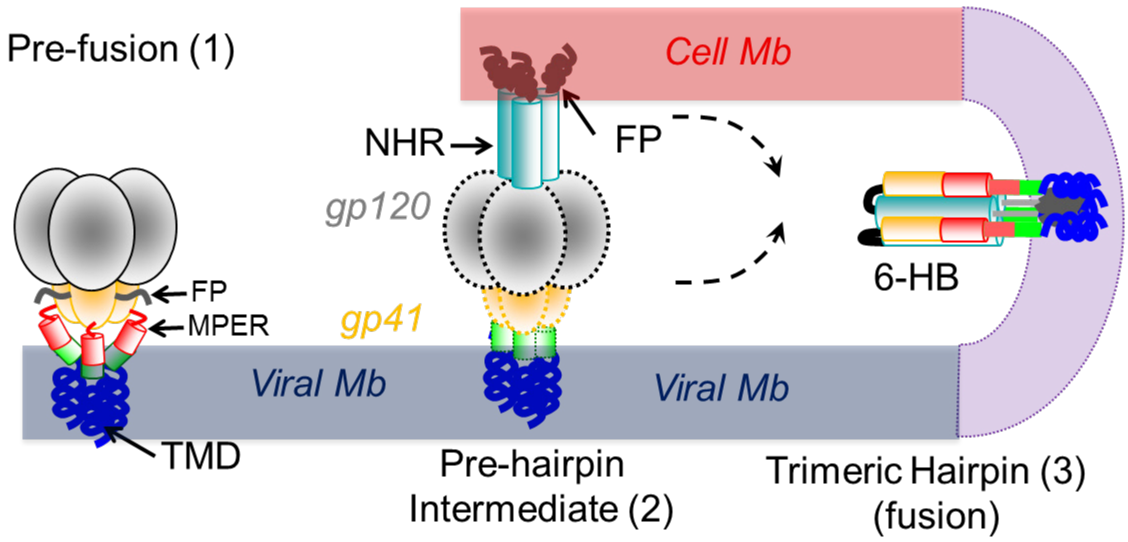

Supplementary Figure S1: General mechanism of HIV-1 Env-promoted fusion. The cartoon highlights three states, namely, 'pre-fusion'(1) (native state in virions, before activation of the process), 'pre-hairpin intermediate'(2) with the FP inserted into the target membrane (assumed to exist soon after fusion activation), and the 'trimeric hairpin' (3), consistent of 6 helices tightly packed into a bundle (6-HB), whose completion is assumed to couple with fusion pore opening (see main text for further explanations). Color code as in Figure 1 of the main text.

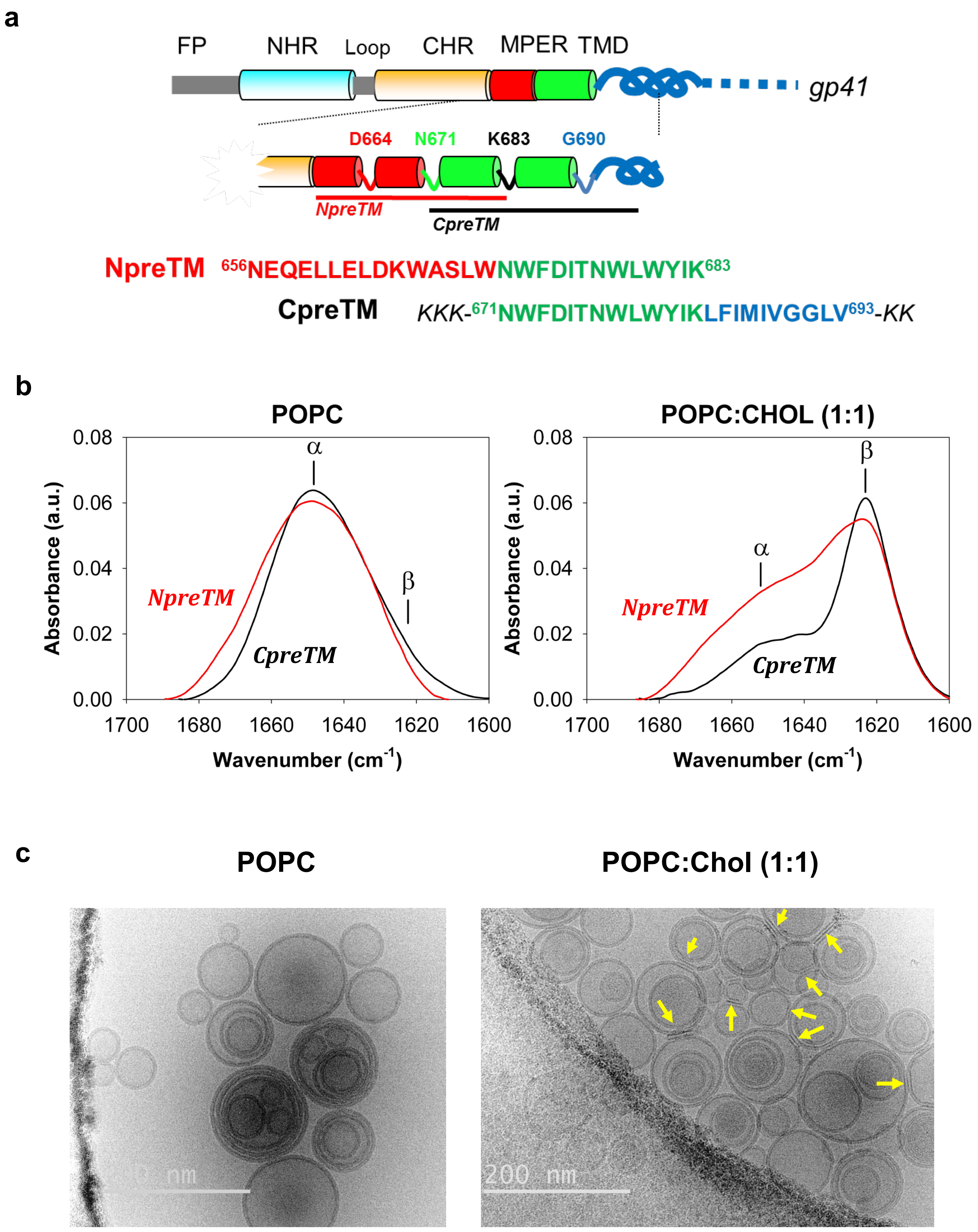
a
FP
NHR
Loop
CHR
MPER
TMD
gp41
D664
N671
K683
G690
NpreTM
CpreTM
NpreTM ⁶⁵⁶NEQELLELDKWASLWNWFDITNWLWYIK⁶⁸³
CpreTM KKK-⁶⁷¹NWFDITNWLWYIKLFIMIVGGLV⁶⁹³-KK
b
POPC
POPC:CHOL (1:1)
Absorbance (a.u.)
Wavenumber (cm-1)
α
β
NpreTM
CpreTM
0.08
0.06
0.04
0.02
0.00
1700
1680
1660
1640
1620
1600
c
POPC
POPC:Chol (1:1)
200 nm

Supplementary Figure S2: Conformations adopted in membranes by the control peptide NpreTM and changes induced on vesicle morphology. (a) Designation of the sequence covered by the NpreTM peptide used in the experiments. **(b)** IR spectra (red lines) in the amide I region obtained after reconstitution of NpreTM in POPC (right) or POPC:Chol 1:1 (left) membranes. Spectra of CpreTM measured under similar conditions have been added for comparison (black lines). **(c)** Cryo-EM images of POPC (right) and POPC:Chol 1:1 (left) vesicles containing NpreTM peptide. Arrows in the POPC:Chol sample indicate flat vesicle-vesicle contacts displaying a trilayer appearance, which are absent in the POPC samples. The peptide-to-lipid mole ratio was 1:50 in panels b and c.

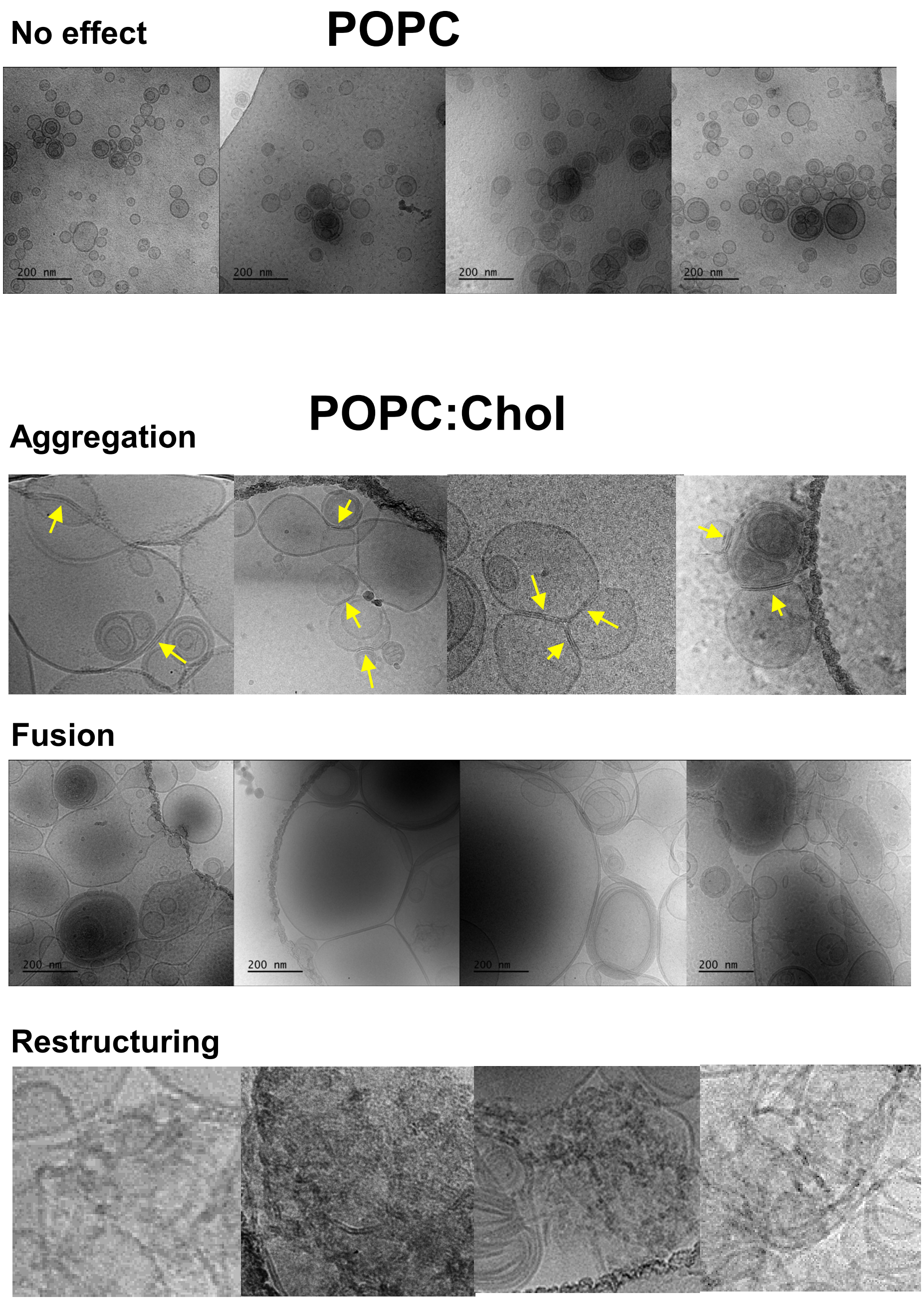

Supplementary Figure S3: Morphological changes induced by CpreTM reconstituted in vesicles of different compositions as determined by cryo-EM. In images illustrating the aggregation pattern, flat zones of tight bilayer-bilayer contact are indicated by the yellow arrows. The peptide was incorporated at a peptide-to-lipid ratio of 1:50 (mol:mol).

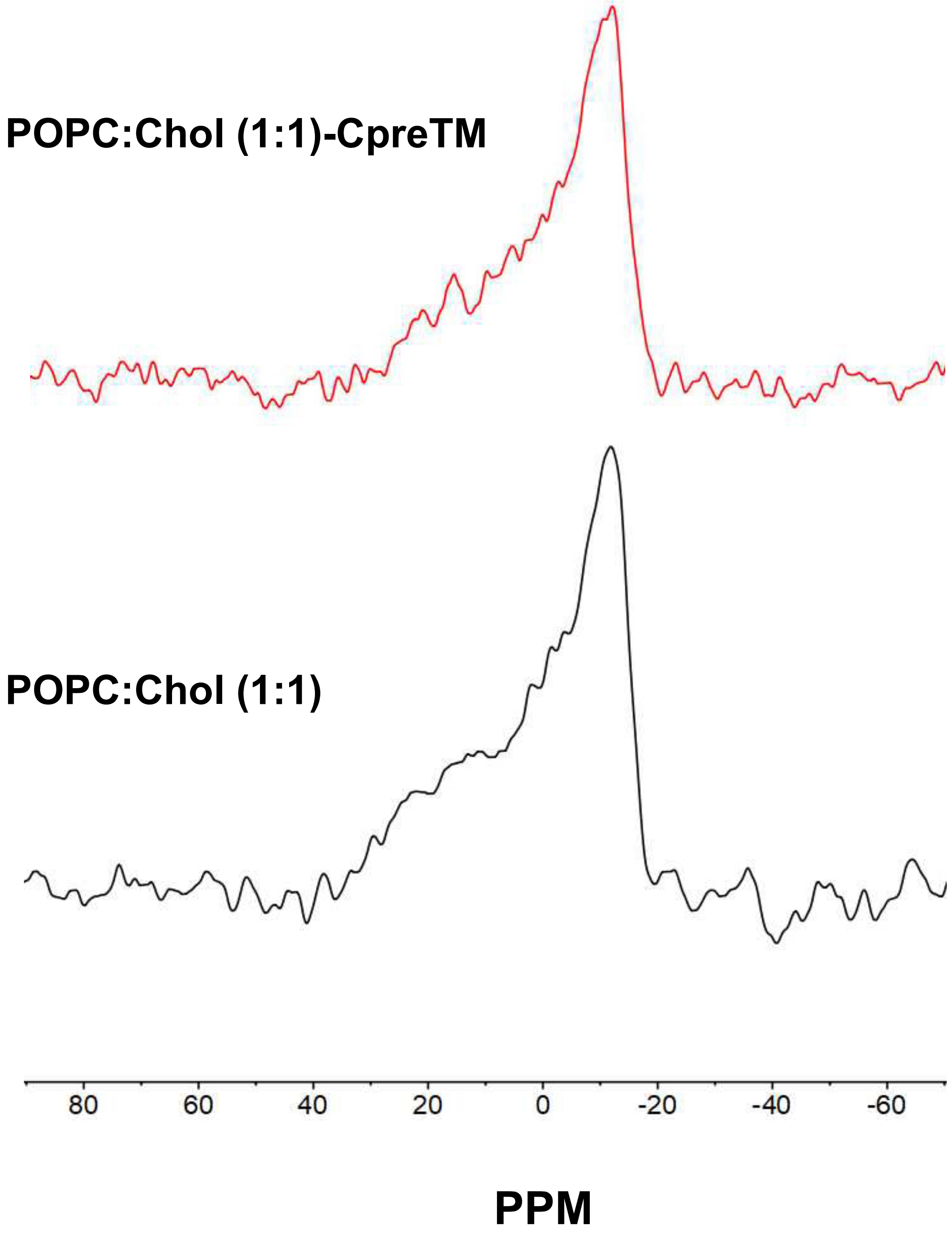

Supplementary Figure 4: ^{31}P-NMR spectra of POPC:Chol (1:1) lipid bilayers without and with CpreTM (black and red spectra, respectively). Both spectra displayed features of the anisotropic lamellar phase (i.e., a high field peak and a low field shoulder), but not isotropic peaks related to the presence of non-lamellar structures in which lipid molecules undergo rapid isotropic motions. The peptide-to-lipid mole ratio was 1:50 (mol:mol) in the sample containing the reconstituted peptide.

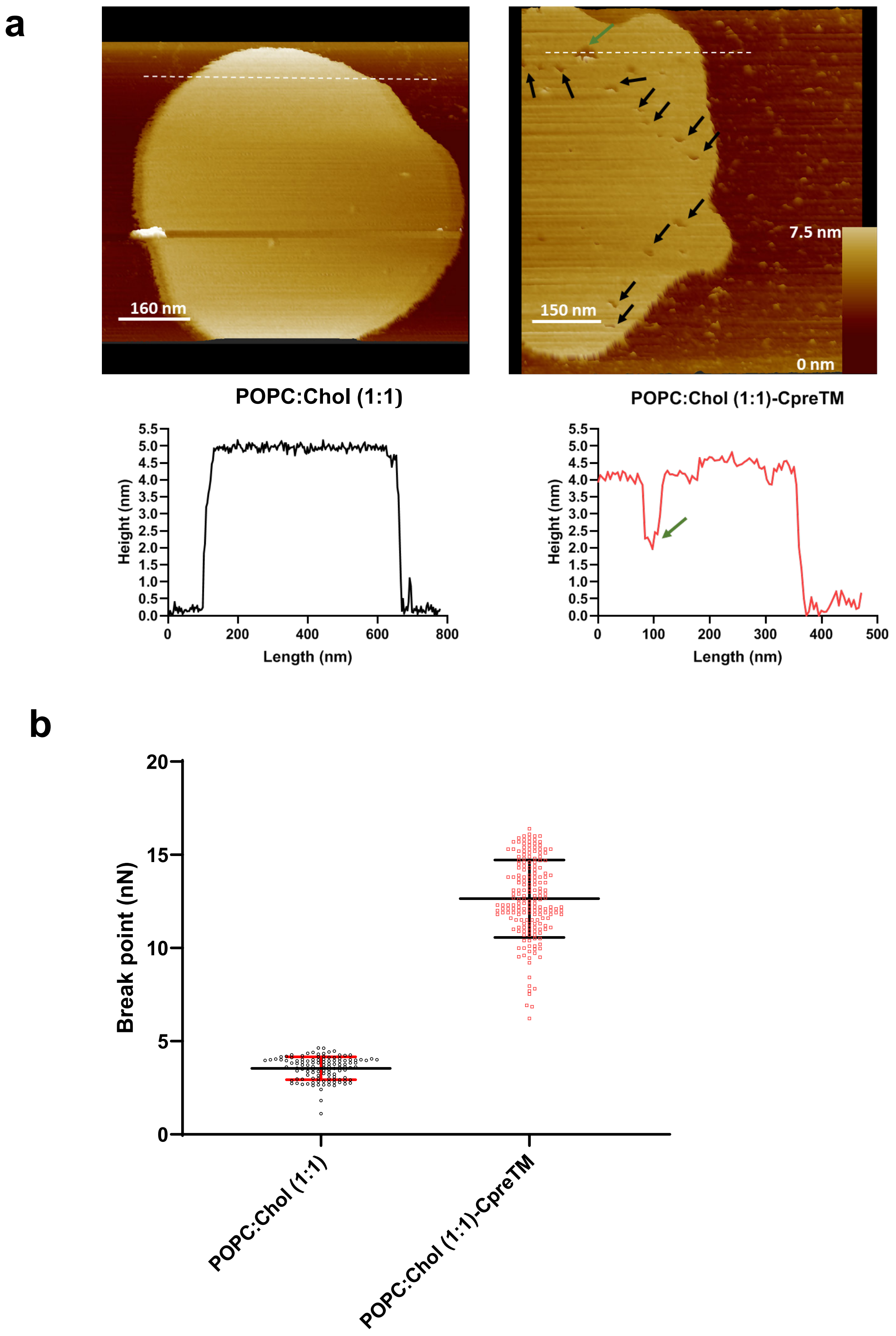
a
160 nm
150 nm
7.5 nm
0 nm
POPC:Chol (1:1)
POPC:Chol (1:1)-CpreTM
Height (nm)
Length (nm)
b
Break point (nN)
POPC:Chol (1:1)
POPC:Chol (1:1)-CpreTM

Supplementary Figure 5: Atomic Force Microscopy characterization of supported lipid bilayers (a) Image and cross section of POPC:Chol (1:1) lipid bilayers without (left) and with CpreTM (right). The presence of the reconstituted peptide modifies the membrane producing holes (black arrows). The cross section, white dotted-line, shows that only the outer layer is affected (green arrow). **(b)** Breakpoint force of POPC:Chol and POPC:Chol + CpreTM bilayers. The control bilayer without peptide shows a significantly lower breakpoint force (3.5455+-0.6151, Mean ±SD) than that containing the reconstituted peptide (12.65+-2.077, Mean ± SD). In both panels the peptide-to-lipid mole ratio was 1:50 in the samples containing CpreTM.

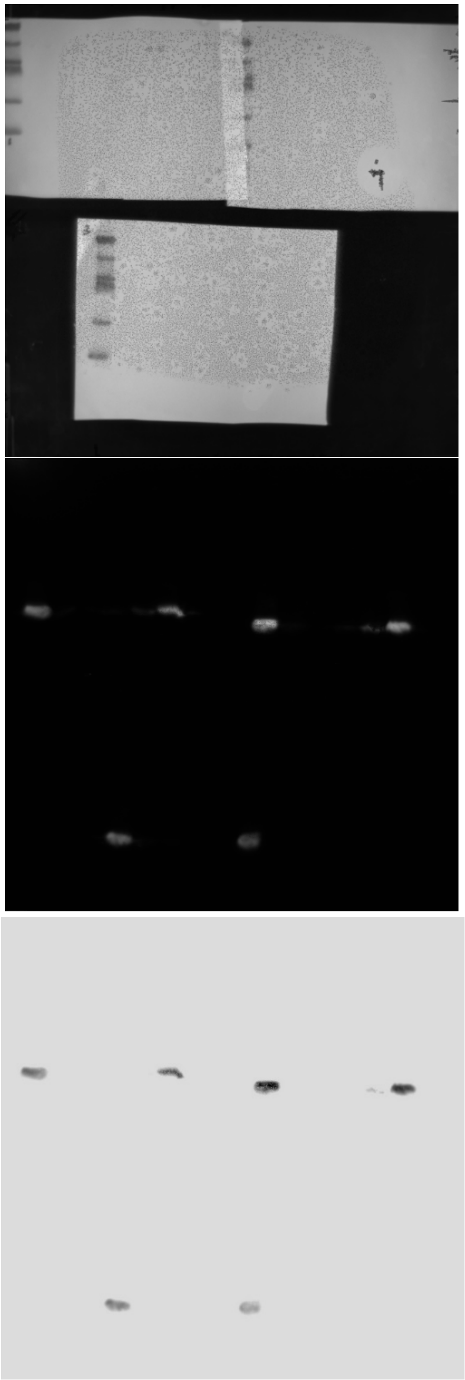

Supplementary Figure S6: Full-length blots used to produce Figure 6a of the main text. Images of the blots displaying single bands for the CpreTM-containing fractions were processed simultaneously.
